# BIMMER: a novel algorithm for detecting differential DNA methylation regions from MBDCap-seq data

**DOI:** 10.1186/1471-2105-15-S12-S6

**Published:** 2014-11-06

**Authors:** Zijing Mao, Chifeng Ma, Tim H-M Huang, Yidong Chen, Yufei Huang

**Affiliations:** 1 Department of Electrical and Computer Engineering, University of Texas at San Antonio, San Antonio, TX 78249-0669, USA; 2 Department of Molecular Medicine, University of Texas Health Science Center at San Antonio, San Antonio, TX 78229, USA; 3 Department of Epidemiology and Biostatistics, University of Texas Health Science Center at San Antonio, San Antonio, TX 78229, USA; 4 Greehey Children's Cancer Research Institute, University of Texas Health Science Center at San Antonio, San Antonio, TX 78229, USA; 5 Cancer Therapy and Research Center, University of Texas Health Science Center at San Antonio, San Antonio, TX 78229, USA

**Keywords:** DNA methylation, differential methylation, MBDCap-seq, Hidden Markov Model (HMM)

## Abstract

DNA methylation is a common epigenetic marker that regulates gene expression. A robust and cost-effective way for measuring whole genome methylation is Methyl-CpG binding domain-based capture followed by sequencing (MBDCap-seq). In this study, we proposed BIMMER, a Hidden Markov Model (HMM) for differential Methylation Regions (DMRs) identification, where HMMs were proposed to model the methylation status in normal and cancer samples in the first layer and another HMM was introduced to model the relationship between differential methylation and methylation statuses in normal and cancer samples. To carry out the prediction for BIMMER, an Expectation-Maximization algorithm was derived. BIMMER was validated on the simulated data and applied to real MBDCap-seq data of normal and cancer samples. BIMMER revealed that 8.83% of the breast cancer genome are differentially methylated and the majority are hypo-methylated in breast cancer.

## Introduction

DNA methylation refers to the chemical modification of DNA nucleotides. One of the most common DNA methylation is the modification of cytosine, which typically occurs in CpG sites. When CpG sites in the promoter region that transcription factors bind are methylated, permanent silencing of gene expression is observed in the cell. DNA methylation is highly prevalent in cancer, involved in almost all types of cancer development by altering the normal regulation of gene expression and silencing the tumor suppressor genes [1]. There are three sequencing-based technologies for whole-genome DNA methylation profiling: bisulfite treatment[2] based or bisulfite sequencing, methylated DNA immunoprecipitation followed by sequencing (MeDIP-seq)[3], and Methyl-CpG binding domain-base capture followed by sequencing (MBDCap-seq)[4]. Among the three technologies, MBDCap-seq has higher dynamic range and better sensitivities and it detects more enrichment in CpG-dense methylated DNA regions [5,6]. We choose to focus on MDBCap-seq data analysis in this study.

Two computational problems concern these genome-wide methylation data including methylation site detection and differential methylation region (DMR) detection. The problem of methylation site detection is similar to the peak detection for ChIP-seq. However, since methylation signals give rise to wider sequence read distribution than that from ChIP-seq peak identification algorithms such as SPP[7] and MACS[8] that are designed primarily for ChIP-seq data analysis would produce poor identification of methylation sites. Specific changes and new algorithms have been proposed to account for the nature of wider read distribution in methylation sequencing data. For instance, Hidden Markov Model [9,10] have been proposed to model the correlation between adjacent bins of a methylation site. The main aim of DMR detection is to identify aberrant DNA methylation regions that are specifically associated with disease phenotype. It is also fundamental to understanding the cause of altered gene expression in cancer. Most of the popular DMR detection pipelines includes two parts: the first part concerns detection of methylation sites in normal and disease samples individually and the second part includes identification of differential methylated regions in disease sample versus normal samples [11]. Many algorithms for differential methylation detection have been proposed including for example ChIPnorm [11] and ChIPDiff [12]. ChIPnorm performs a quantile normalization on normal and disease samples and applies differential analysis to detect DMRs, whereas ChIPDiff detects enriched methylation regions in normal and disease samples with a Binomial model, and then performs differential analysis based on a HMM. These existing algorithms are very powerful tools in differential methylation analysis but they have clear disadvantages especially when applied to MBDCap-seq. First, the targeted resolutions of the data are relatively low, for instance, the bin size in ChIPDiff is 1000 base pairs (bps). However for a typical MBDCap-seq data, the resolution is normally 100bp bins. Second, these existing pipelines mentioned above are two-step procedures, which are prone to error propagation. If there is an error in methylation site detection, this error will be passed on to the following DMR detection step and impact negatively the performance of differential methylation. Last but not the least, with an exception in [13], most existing algorithms were developed to handle single sample. When replicates are available, they perform prediction on individual samples separately and then fuse the detection results from individual together. Such fusion based algorithms is easily influenced by the erroneous predictions made at individual level. The algorithm in [13] applies LOESS to normalize the difference between replicate samples. However, it assumes that only a small portions of methylation regions are DMRs [11], which might not be applicable to all cases.

In this paper, we proposed a novel algorithm for differential methylation regions (DMRs) detection based on Hidden Markov Model (HMM) and we call the algorithm BIMMER. BIMMER models the methylation status and detect DMRs in normal and cancer samples simultaneously. By doing this, BIMMER avoids error propagation in the existing two-step pipelines and therefore can improve the performance of DMRs detection. BIMMER was tested first on a simulated datasets and applied to a real breast cancer MBDCAP-seq data. The results from breast cancer data revealed that there are 8.83% of 30,804,183 bines detected with differentially methylated status, most of which are hypo-methylated in breast cancer samples.

## Methods

### Notation

Each MBDCap-seq data sample is pre-processed to be in a BED file, which records the sequence reads counts in consecutive 100 base pair (bp) bins over the entire genome. Let's denote the sample size of the normal sample MBDCap-Seq datasets as $N_{1}$, that of the cancer dataset as $N_{2}$, and the total number of the bins is denoted as  M. We further denote the reads count of the $i_{th}$ bin in $N_{1}$ normal samples by a vector$X_{n_{i}}={\left[X_{n_{i}.1},X_{n_{i}.2},\ldots ,X_{n_{i}.N_{1}}\right]}^{⊤}$, where $X_{n_{i},j}$ is the reads count of the $j_{th}$ sample, and similarly the reads count of the $i_{th}$ bin in $N_{2}$ cancer samples by $X_{c_{i}}={\left[X_{c_{i}.1},X_{c_{i}.2},\ldots ,X_{c_{i}.N_{2}}\right]}^{⊤}$, where $X_{c\text{\_}i,k}$ represents the reads count in the $k_{th}$ sample. The aim of this work is to predict the differential methylation status of the cancer samples over the normal samples for every bin in the genome.

### Two layer HMM model for differential methylation

**Figure 1:**
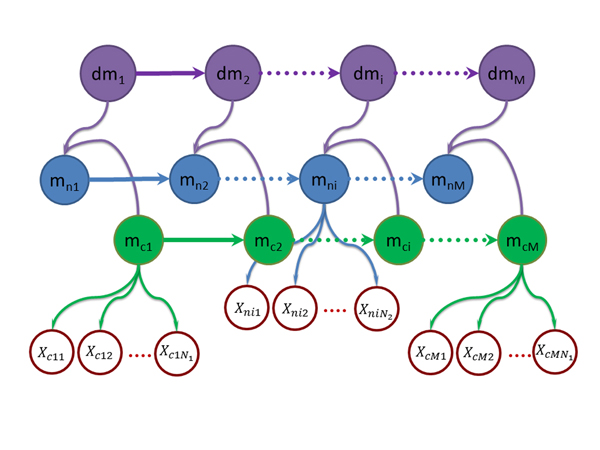
**The proposed bi-layer HMM for differential methyulation analysis. **The first layer HMMs model the methylation statuses in the normal and disease samples in the bins, where $X_{c}$'s and $m_{c}$'s represent the reads counts and the methylations status in the normal samples, respectively, and $X_{n}$'s and $m_{n}$'s those in the cancer samples. The second layer HMM models the relationship between the differntial methylation status, *dm*, and the methylations statuses of $m_{n}$ and *$m_{c}$*.

A bin is considered differential methylated if its methylation status in the cancer sample is different from that in the normal samples. Therefore, the methylation models for the $N_{1}$ normal samples and the $N_{2}$ cancer samples needs to be defined before proceeding to model the differential methylation. To model the methylation status, let $m_{n_{i}}=\left[0,1\right]$ denote the methylation status of the $i_{th}$ bin for the normal sample, where $m_{n_{i}}=1$ when the $i_{th}$ bin is methylated and $m_{n_{i}}=0$ otherwise. Because the methylation statuses in the adjacent binds are highly correlated, a first order Markov chain is introduced (Figure 1), where the transition probability is defined as $A_{n}\left(i\right)=p\left(m_{n_{i}}\text{|}m_{n_{i-1}}\right)$ and the initial probability for bin 1 is defined as $\tau_{n}\left(i\right)=P\left(m_{n_{1}}\right)$. Then for each bin, the read counts would depend on the methylation status, which is modeled as an i.i.d discrete distribution as $B_{n}\left(r_{j},d\right)=\overset{N_{1}}{\underset{j=1}{\prod }}P\left(x_{n_{j}}=r_{j}|m_{n_{j}}=d\right).$ Taken together, the methylation in the normal samples is modeled by an HMM. Similarly, the methylation status for the cancer samples can be also modeled by an HMM. Specifically, if let $m_{c_{i}}=\left[0,1\right]$ denote the methylation status of the $j_{th}$ bin of the cancer samples, the transition probability and the initial state probability are modeled as $A_{c}\left(i\right)=p\left(m_{c_{i}}\text{|}m_{c_{i}-1}\right)$ and $\tau_{c}\left(i\right)=P\left(m_{c_{1}}\right),$ respectfully and the emission probability is represented as $B_{c}\left(r_{k},d\right)=\overset{N_{2}}{\underset{k=1}{\prod }}P\left(x_{c_{k}}=r_{k}|m_{c_{k}}=d\right).$ Next, let differential status at the $i_{th}$ bin denoted by $dm_{i}=\left[0,1\right]$, where $dm_{i}=\left\{\begin{array}{c} 1,when\;m_{n_{i}}\neq m_{c_{i}}\\ 0,otherwise \end{array}\right)$. Because the differential methylation statuses for the adjacent bins are also correlated, $dm_{i}$ is further assumed to follow another first order Markov chain (Figure 1), whose transition probability and initial state probability are defined as $A_{dm}\left(i\right)=p\left(dm_{i}\text{|}dm_{i-1}\right)$ and $\tau_{dm}\left(i\right)=P\left(dm_{i}\right)$. Finally, we need to model the relationship between the differential methylation status $dm_{i}$ and the methylation statuses $m_{n_{i}}$ and $m_{c_{i}}$. In this work, we propose to model it as depicted in Figure 1 by the emission probability $P\left(m_{n_{i}}|m_{n_{i-1}},m_{c_{i}},dm_{i}\right)$, i.e., the normal sample methylation status depends directly on the cancer sample methylation status and the differential methylation status, in addition to its own correlations between adjacent bins. It is easy to see from Figure 1 that there are two sets of relatively well defined relationships involved in this emission probability: $p\left(m_{n_{i}}\text{|}m_{n_{i-1}}\right)$ and $P\left(m_{n_{i}}\text{|}m_{c_{i}},dm_{i}\right)$. The first one $p\left(m_{n_{i}}\text{|}m_{n_{i-1}}\right)$ is the transition probability for the methylation status in normal sample and the second one$P\left(m_{n_{i}}\text{|}m_{c_{i}},dm_{i}\right)$ models the dependence of $m_{n_{i}}$ on $m_{c_{i}}$and$dm_{i}$, which can be intuitively defined as $P\left(m_{n_{i}}\text{|}m_{c_{i}},dm_{i}\right)=0,\;if\;m_{n_{i}}=m_{c_{i}}\;but\;dm_{i}=1,\;or\;m_{n_{i}}\neq m_{c_{i}}\;but\;dm_{i}=0$ and $P\left(m_{n_{i}}\text{|}m_{c_{i}},dm_{i}\right)=1,\;otherwise$, i.e., $m_{n_{i}}$ and $m_{c_{i}}$have to be different if $dm_{i}=1$and otherwise they must be the same. Now, the question is how to integrate $p\left(m_{n_{i}}\text{|}m_{n_{i-1}}\right)$ and $P\left(m_{n_{i}}\text{|}m_{c_{i}},dm_{i}\right)$ to model the emission probability$P\left(m_{n_{i}}\text{|}m_{n_{i-1}},m_{c_{i}},dm_{i}\right)$. To this end, a popular approach in data fusion is adopted, which combines them through a weighted sum: $P\left(m_{n_{i}}\text{|}m_{n_{i-1}},m_{c_{i}},dm_{i}\right)=\alpha p\left(m_{n_{i}}\text{|}m_{n_{i-1}}\right)+\left(1-\alpha \right)P\left(m_{n_{i}}\text{|}m_{c_{i}},dm_{i}\right)$ where α is the weighting factor to be determined from data. Taken together, we propose a two-layer HMM model as depicted in Figure 1 for differential methylation and we refer this model as BIMMER. With BIMMER, the differential methylation status is predicted according to the posterior distribution$P\left(dm_{i}|X_{n_{i}},X_{c_{i}}\forall i\right)$. This posterior distribution does not depend on the hard decisions on the methylation states for the normal and cancer samples and therefore overcomes the aforementioned problems of error propagation. However, one difficulty is that in the calculation of this posterior distribution, we need to calculate the integration of all 9 model parameters: $\tau_{n}$, $\tau_{c}$, $B_{n}$,$B_{c}$, $A_{n}$,$A_{c}$,$\tau_{dm}$, $A_{dm}\;and\;\alpha$, which is analytically intractable. To solve this problem, we propose the next Expectation and Maximization (EM) solution.

### The EM solution

Let $X_{n}$ denote the collection of the reads counts in all *M *bins for all $N_{1}$ normal samples and $X_{c}$ the collection of reads counts in all *M *bins for all $N_{2}$ cancer samples. Also, let $m_{n}={\left[m_{n1},m_{n2},\ldots m_{ni}\right]}^{T}$, $m_{c}={\left[m_{c1},m_{c2},\ldots m_{ci}\right]}^{T}$ and $dm={\left[dm_{1},dm_{2},\ldots dm_{i}\right]}^{T}$. In order to obtain the EM solution, $X_{n}$ and $X_{c}$ are treated as the observed data but $m_{n}$ and $m_{c}$ are considered as the unobserved data for the first layer HMM whiledm is the unobserved data for the second layer HMM. Here,  Ψ is used to denote the model parameter set. For the simplicity of the computation, the first layer HMM parameters$\tau_{n}$,$\tau_{c}$, $B_{n}$,$B_{c}$, $A_{n}$,$A_{c}$ are learned directly from $X_{n}$ and $X_{c}$ with Baum-Welch algorithm and excluded from the EM process. Therefore, the parameter set  Ψ for BIMMER includes 3 parameter: $\Psi =\left\{\tau_{dm},\;A_{dm},\alpha \right\}$. Given a set of initial or estimated parameters, the complete data likelihood function is

$$\begin{array}{c} L\left(\Psi \right)=P\left(X_{n},X_{c},m_{c},m_{n},dm|\Psi \right)\\ \;\;\;=\tau_{dm}\times \tau_{c}\times \left(\alpha \times \tau_{n}+\left(1-\alpha \right)\times P\left(m_{n1}|m_{c1},dm_{1},\Psi \right)\right)\times P\left(x_{n1}|m_{n1},\Psi \right)\times P\left(x_{c1}|m_{c1},\Psi \right)\\ \;\;\;\times \overset{M}{\underset{i=2}{\prod }}[P\left(x_{ni}|m_{ni},\Psi \right)\times P\left(x_{ci}|m_{ci},\Psi \right)\times P\left(m_{ci}|m_{c\left(i-1\right)},\Psi \right)\times P\left(m_{ni}|m_{n\left(i-1\right)},m_{ci},dm_{i},\Psi \right)\\ \;\;\;\times P\left(dm_{i}|dm_{i-1},\Psi \right)] \end{array}$$

Then the log-likelihood function can be expressed as

$$\begin{array}{c} \text{log}\left(l\left(\Psi \right)\right)=\text{log}\left(\tau_{dm}\right)\\ \;\;\;\;\;\;\;+\text{log}\left(\tau_{c}\right)+\text{log}\left(\alpha \times \tau_{n}+\left(1-\alpha \right)\times P\left(m_{n1}\text{|}m_{c1},dm_{1},\Psi \right)\right)+\text{log}\left(P\left(x_{n1}\text{|}m_{n1},\Psi \right)\right)+\text{log}\left(P\left(x_{c1}\text{|}m_{c1},\Psi \right)\right)\\ \;\;\;\;\;\;\;+\overset{M}{\underset{i=2}{\sum }}[\text{log}\left(P\left(x_{ni}\text{|}m_{ni},\Psi \right)\right)+\text{log}\left(\left(x_{ci}\text{|}m_{ci},\Psi \right)\right)+\text{log}\left(P\left(m_{ci}\text{|}m_{c\left(i-1\right)},\Psi \right)\right)\\ \;\;\;\;\;\;\;+\text{log}\left(P\left(m_{ni}\text{|}m_{n\left(i-1\right)},m_{ci},dm_{i},\Psi \right)\right)+\text{log}\left(P\left(dm_{i}\text{|}dm_{i-1},\Psi \right)\right)] \end{array}$$

At the $k_{th}$ iteration, suppose that the estimated parameter set at the previous iteration is $\Psi^{k-1}$. Then, at E-step, the conditional expectation of this log complete data likelihood is calculated

$$Q\left(\Psi ;\Psi^{k-1}\right)=E_{\Psi^{k-1}}\left[logL\left(\Psi \right)\text{|}X_{n},X_{c}\right]=\underset{m_{c}}{\sum }\underset{m_{n}}{\sum }\underset{dm}{\sum }\overset{M}{\underset{i=1}{\sum }}logP\left(X_{n},X_{c},m_{ci},m_{ni},dm_{i}|\Psi \right)\overset{M}{\underset{i=1}{\prod }}P\left(m_{ci},m_{ni},dm_{i}\text{|}X_{n},X_{c},\Psi^{k-1}\right)$$

In order to obtain$P\left(m_{ci},m_{ni},dm_{i}\text{|}X_{n},X_{c},\Psi^{k-1}\right)$, the forward-backward algorithm is used, where

$$\begin{array}{c} P\left(m_{ci},m_{ni},dm_{i}\text{|}X_{n},X_{c},\Psi^{k-1}\right)\propto P\left(dm_{i},m_{ci},m_{ni},X_{n},X_{c}|\Psi^{k-1}\right)\\ \;\;\;\;\;\;=P\left(dm_{i},m_{ci},m_{ni},X_{n,1:i},X_{c,1:i}|\Psi^{k-1}\right)\times P\left(X_{n,i+1:M},X_{c,i+1:m}\text{|}dm_{i},m_{ci},m_{ni},X_{n,},X_{c,1:M},\Psi^{k-1}\right)\\ \;\;\;\;\;\;=P\left(dm_{i},m_{ci},m_{ni},X_{n,1:i},X_{c,1:i}|\Psi^{k-1}\right)\times P\left(X_{n,i+1:M},X_{c,i+1:m}\text{|}dm_{i},m_{ci},m_{ni},\Psi^{k-1}\right) \end{array}$$

$$P\left(dm_{i},m_{ci},m_{ni},X_{n,1:i},X_{c,1:i}|\Psi^{k-1}\right)=\overset{}{\underset{dm_{i-1}}{\sum }}\overset{}{\underset{m_{c\left(i-1\right)}}{\sum }}\overset{}{\underset{m_{n\left(i-1\right)}}{\sum }}P\left(dm_{i},dm_{i-1},m_{ci},m_{c\left(i-1\right)},m_{ni},m_{n\left(i-1\right)},X_{n,1:i-1},X_{c,1:i-1},|\Psi^{k-1}\right)$$

and

$\begin{array}{c} P\left(X_{n,i+1:M},X_{c,i+1:m}\text{|}dm_{i},m_{ci},m_{ni},\Psi^{k-1}\right)=\\ \overset{}{\underset{dm_{i-1}}{\sum }}\overset{}{\underset{m_{c\left(i-1\right)}}{\sum }}\overset{}{\underset{m_{n\left(i-1\right)}}{\sum }}P\left(dm_{i+1},m_{n\left(i+1\right)},m_{c\left(i+1\right)},X_{n,i+1:M},X_{c,i+1:M}|dm_{i},m_{ci},m_{ni},\Psi^{k-1}\right) \end{array}$.

where $X_{n,p:q}$ and $X_{c,p:q}$ denote the collection of the reads counts from bin *p *to bin *q *from the *N*_1 _normal samples and the *N*_2 _cancer samples, respectively. In the forward step, we calculate

$$\begin{array}{c} P\left(dm_{i},dm_{i-1},m_{ci},m_{c\left(i-1\right)},m_{ni},m_{n\left(i-1\right)},X_{n,1:i-1},X_{c,1:i-1}|\Psi^{k-1}\right)\\ \;\;\;\;\;\;=P\left(dm_{i},dm_{i-1},m_{ci},m_{c\left(i-1\right)},m_{ni},m_{n\left(i-1\right)},X_{n,1:i-1},X_{c,1:i-1}|\Psi^{k-1}\right)\\ \;\;\;\;\;\;\times P\left(m_{ci}\text{|}dm_{i},dm_{i-1},m_{n\left(i-1\right)},m_{c\left(i-1\right)},X_{n,1:i-1},X_{c,1:i-1},\Psi^{k-1}\right)\\ \;\;\;\;\;\;\times P\left(dm_{i}\text{|}dm_{i-1},m_{n\left(i-1\right)},m_{c\left(i-1\right)},X_{n,1:i-1},X_{c,1:i-1},\Psi^{k-1}\right)\\ \;\;\;\;\;\;\times P\left(dm_{i-1},m_{n\left(i-1\right)},m_{c\left(i-1\right)},X_{n,1:i-1},X_{c,1:i-1}|\Psi^{k-1}\right) \end{array}$$

$=P\left(X_{ni}|m_{ni},\Psi^{k-1}\right)\times P\left(X_{n,i+2:M},X_{c,i+2:m}\text{|}dm_{i+1},m_{c\left(i+1\right)},m_{n\left(i+1\right)},\Psi^{k-1}\right)$.

In the backward step, we have

$$\begin{array}{c} P\left(dm_{i+1},m_{c\left(i+1\right)},m_{n\left(i+1\right)},X_{n,i+1:M},X_{c,i+1:M}|dm_{i},m_{ci},m_{ni},\Psi^{k-1}\right)\\ \;\;\;\;\;\;=P\left(X_{n\left(i+1\right)}|dm_{i},dm_{i+1},m_{ci},m_{c\left(i+1\right)},m_{ni},m_{n\left(i+1\right)},X_{n,i+2:M},X_{c,i+2:M},\Psi^{k-1}\right)\\ \;\;\;\;\;\;\times P\left(X_{c\left(i+1\right)}|dm_{i},dm_{i+1},m_{ci},m_{c\left(i+1\right)},m_{ni},m_{n\left(i+1\right)},X_{n,i+2:M},X_{c,i+2:M},\Psi^{k-1}\right)\\ \;\;\;\;\;\;\times P\left(m_{n\left(i+1\right)}|dm_{i},dm_{i+1},m_{ci},m_{c\left(i+1\right)},m_{ni},X_{n,i+2:M},X_{c,i+2:M},\Psi^{k-1}\right)\\ \;\;\;\;\;\;\times P\left(m_{c\left(i+1\right)}|dm_{i},dm_{i+1},m_{ci},m_{ni},X_{n,i+2:M},X_{c,i+2:M},\Psi^{k-1}\right)\times P\left(dm_{i+1}|dm_{i+2},\Psi^{k-1}\right)\\ \;\;\;\;\;\;\times P\left(X_{n,i+2:M},X_{c,i+2:M}|dm_{i+1},m_{c\left(i+1\right)},m_{n\left(i+1\right)},\Psi^{k-1}\right) \end{array}$$

Then, at M-step, the parameter set $\Psi^{k}$ is updated from $\Psi^{k-1}$ by maximizing the likelihood expectation with respect to $\Psi^{k-1}$. This process is equivalent to maximizing he  Q function with respective to the parameters  Ψ

$$\Psi^{k}=argmax_{\Psi }Q\left(\Psi ;\Psi^{k-1}\right)$$

The maximization yields

$\tau_{dm}\left(z\right)=\overset{}{\underset{m_{c1}}{\sum }}\overset{}{\underset{m_{n1}}{\sum }}P\left(dm_{1}=z,m_{c1},m_{n1},X_{ni},X_{ci}|\Psi^{k-1}\right)$,

$$A_{dm}\left(i\right)=P\left(dm_{i}\text{|}dm_{i-1},\Psi^{k-1}\right)=\frac{P\left(dm_{i},dm_{i-1,}X_{ni},X_{ci}|\Psi^{k-1}\right)}{P\left(dm_{i-1,}X_{ni},X_{ci}|\Psi^{k-1}\right)}$$

where

$$\begin{array}{c} P\left(dm_{i},dm_{i-1,}X_{ni},X_{ci}|\Psi^{k-1}\right)\\ \;\;\;\;\;=\underset{m_{ci}}{\sum }\underset{m_{ni}}{\sum }\underset{m_{n\left(i-1\right)}}{\sum }\underset{m_{c\left(i-1\right)}}{\sum }P\left(X_{ni}|m_{ni},\Psi^{k-1}\right)P\left(X_{ci}|m_{ci},\Psi^{k-1}\right)P\left(X_{n,i+1:M},X_{c,i+1:M}|dm_{i},m_{ci},m_{ni},\Psi^{k-1}\right)\\ \;\;\;\;\;\times P\left(m_{ni}|dm_{i},m_{ci},m_{ni},m_{n\left(i-1\right)},\Psi^{k-1}\right)P\left(m_{ci}|m_{c\left(i-1\right)},\Psi^{k-1}\right)P\left(dm_{i}|dm_{i-1},\Psi^{k-1}\right)\\ \;\;\;\;\;\times P\left(dm_{i-1},m_{ci-1},m_{ni-1},X_{n1:i-1},X_{c1:i+1}|\Psi^{k-1}\right) \end{array}$$

and

$$\begin{array}{c} P\left(dm_{i-1,}X_{ni},X_{ci}|\Psi^{k-1}\right)\\ =\overset{}{\underset{m_{ni-1}}{\sum }}\overset{}{\underset{m_{ci-1}}{\sum }}P\left(X_{n,i-1:M},X_{c,i-1:M}|dm_{i-1},m_{c\left(i-1\right)},m_{n\left(i-1\right)},\Psi^{k-1}\right)P\left(dm_{i-1},m_{c\left(i-1\right)},m_{n\left(i-1\right)},X_{n,1:i-1},X_{c,1:i+1}|\Psi^{k-1}\right) \end{array}$$

and

$$\begin{array}{c} \frac{\partial \sum_{m_{c},m_{n},dm}log\left(P\left(X_{c},X_{n},m_{c},m_{n},dm|\Psi \right)P\left(X_{c},X_{n},m_{c},m_{n},dm|\Psi^{k-1}\right)\right)}{\partial \alpha }\\ \;\;\;\;=\overset{M}{\underset{i=2}{\sum }}\underset{m_{ci}}{\sum }\underset{m_{ni}}{\sum }\underset{dm_{i}}{\sum }\underset{m_{ni-1}}{\sum }\frac{P\left(m_{ci},m_{ni},dm_{i},m_{ni-1},X_{c},X_{n},|\Psi^{k-1}\right)}{\alpha +\frac{P\left(m_{ni}|m_{ci},dm_{i},\Psi^{k-1}\right)}{P\left(m_{ni}|m_{n\left(i-1\right)},\Psi^{k-1}\right)-P\left(m_{ni}|m_{ci},dm_{i},\Psi^{k-1}\right)}}\\ \;\;\;\;+\underset{m_{c1}}{\sum }\underset{m_{n1}}{\sum }\underset{dm_{1}}{\sum }\frac{P\left(m_{c1},m_{n1},dm_{1},X_{c},X_{n},|\Psi^{k-1}\right)}{\alpha +\frac{P\left(m_{1}|m_{1},dm_{1},\Psi^{k-1}\right)}{\tau_{c}-P\left(m_{n1}|m_{c1},dm_{1},\Psi^{k-1}\right)}} \end{array}$$

where the last equation is calculated by the Newton-Raphson algorithm. Maximizing this  Q function guarantees that the likelihood $L\left(\Psi^{k}\right)$ is always greater than $L\left(\Psi^{k-1}\right)$, hence ensures global convergence of the solution.

### Model initialization and prediction of DMRs

To implement the EM algorithm, the initial parameter set Φ^(0) ^and the parameters for the first layer needs to be carefully defined because specific choice of these initial parameter values could lead to difference local optimal solutions and affect the prediction performance. After the convergence of the EM solution, the differential methylation statuses, **dm**, are predicted using the Viterbi algorithm[14] as the chain of the states with the largest probability given the estimated parameter set. Additionally, the methylation statuses $m_{c}\;\text{and}\;m_{n}$ can also be predicted using the Viterbi algorithm provided the parameters of the first layer HMM are set to the estimated ones.

## Results

BIMMER was validated on both simulated data and applied to a real breast cancer dataset. It was first tested on the simulated systems, where the data models were assumed known. Then, BIMMER was applied to a real breast cancer dataset to explore the state of differential methylation.

### Test on simulated data

**Figure 2:**
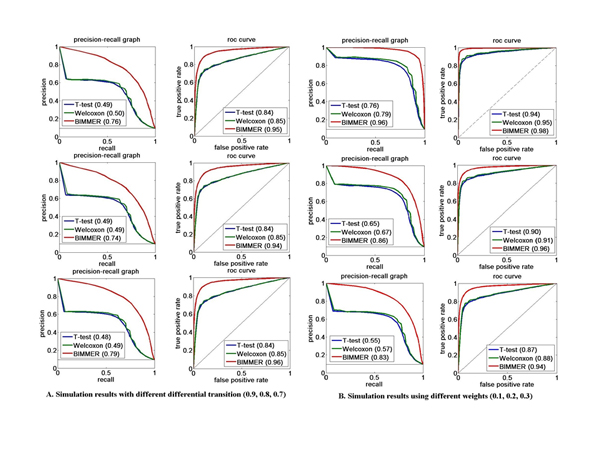
**The performance of BIMMER on Simulated Data**. A The pricision-recall curves and the ROC curves for different transitional probabiliteis of differential methylation status $P\left(dm_{i}=1\text{|}dm_{i-1}=1\right)$. The top row for $P\left(dm_{i}=1\text{|}dm_{i-1}=1\right)=0.9$, the middle row for $P\left(dm_{i}=1\text{|}dm_{i-1}=1\right)=0.8$, and the bottom row for $P\left(dm_{i}=1\text{|}dm_{i-1}=1\right)=0.7$. B. The pricision-recall curves and the ROC curves for different weights. The top row for α=0.1, the middle row for α=0.2, and the bottom row for α=0.3.

**Figure 3:**
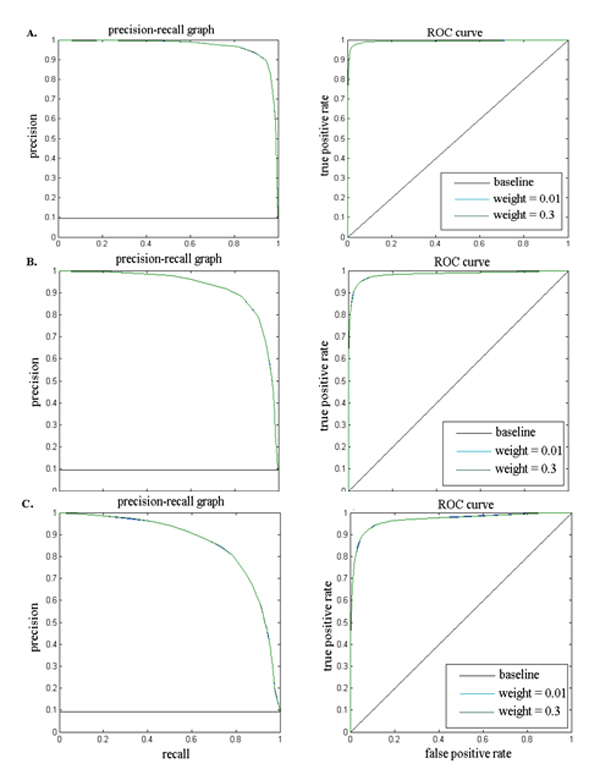
**The performance of BIMMER for different initial weights (0.01, 0.3).** A. Results for the true weight *ω *= 0.1; B. Results for the true weight *ω *= 0.2; C. Results for the true weight *ω *= 0.3.

A test dataset was simulated based on the graphical model in Figure 1 to evaluate the performance of BIMMER. A chain of **dm **was first generated based on given $\tau_{dm}$ and $A_{dm}$. The methylation status in normal and cancer sample $m_{c}$ and $m_{n}$ were then generated based on a set of $\tau_{c}$, $\tau_{n},\;A_{c}$, $A_{n}$ and weight parameter  α. The read counts in each bin of the normal and cancer sample $X_{c}$ and $X_{n}$ were generated according to the emission probabilities $B_{n}$ amd $B_{c}$. In addition, a Poisson noise was also added to the reads. Multiple sets of 10 samples with 200,000 bins were generated with different transition probabilities and weight factors (See Table 1 for detailed parameter settings). For comparison, two commonly used differential analysis algorithms: two sample *t*-test and Wilcoxon Test [15,16] were also applied to the simulation data. To test the performance of BIMMER under different conditions, two scenarios were considered, where in the first all the parameters except the transition probabilities were fixed, whereas in the other situation only the weight factors were allowed to change. The prediction results were evaluated by the precision and recall (PR) curve and receiver operating characteristic (ROC) curve while the area under the curve (AUC) were calculated for each algorithm (Figure 2). For all the simulation tests, BIMMER outperformed both the two sample t-test and Wilcoxon test. In each simulated scenario, the performance of Wilcoxon test and the two sample *t*-test were very similar. The Wilcoxon slightly outperformed the *t*-test because the Wilcoxon test is more robust. For the scenario, the transition probabilities of the simulation were sets from 0.9 to 0.7 and the performance of BIMMER did not change much in terms of its PR and ROC curve. For the second scenario, as the weight factors increasing, the performance of BIMMER slightly decreases. This makes sense because the weight factors actually models the contribution factors of two probabilities $p\left(m_{n_{i}}\text{|}m_{n_{i-1}}\right)\;and\;P\left(m_{n_{i}}\text{|}m_{c_{i}},dm_{i}\right)$. The larger this weight factors is, the more uncertainty exist. As the result, the prediction performance decrease. We further tested the influence of different initial values of the weight on the final prediction of differential methylation in the EM solution. This requires to be tested because  α is unique in our model. Different initial weights (0.01 and 0.3) were tested used in three simulations and the prediction performance of BIMMER (Figure 3) showed little difference, indicating that the initial *ω *has little influence on BIMMER's prediction results. (The simulation transition probability and the training result are provided in Table 2) In conclusion, BIMMER can produce satisfactory prediction results on the simulation data; it is also robust against changes in the transition probabilities and the weights.

**Table 1:** Parameter set used for simulation.

Table 1-1						
**$\tau_{c}$**	**$A_{c}$**		**Likelihood Of $m_{c}$**			

0.99999	0.7296	0.2704	-1.619118E7			
0.00001	0.0225	0.9775				

**Table 1-2**						

**$\tau_{n}$**	**$A_{n}$**		**Likelihood Of $m_{n}$**			

0.99999	0.7614	0.2386	-2.928487E7			
0.00001	0.0563	0.9437				

**Table 1-3**						

**Symbols **	**0**	**1**	**2**	**3**	**4**	**5**

$m_{c}$ = 0	0.9	0.04	0.03	0.01	0.01	0.01
$m_{c}$ = 1	0.26	0.24	0.2	0.18	0.08	0.04

**Table 1-4**						

**Symbols **	**0**	**1**	**2**	**3**	**4**	**5**

$m_{n}$ = 0	0.8	0.08	0.07	0.03	0.01	0.01
$m_{n}$ = 1	0.22	0.26	0.20	0.16	0.1	0.06

**Table 1-5**						

$\tau_{dm}$	$A_{dm}$		Weight α	**Likelihood Of dm**		

0.99999	0.9705	0.0295	0.3519	-4.538562E8		
0.00001	0.2862	0.7138				

**Table 2:** Estimated parameters after training using different initial weight: 0.01 and 0.3

Weight of simulator		0.3		0.2		0.1	
Initial weight for training		0.01	0.3	0.01	0.3	0.01	0.3
Transition of simulator	Weight	0.2820	0.2850	0.1993	0.2002	0.0974	0.0980
0.97	Differential Transition	0.9705	0.9710	0.9698	0.9699	0.9689	0.9690
0.71		0.7146	0.7173	0.7186	0.7192	0.7048	0.7044
0.76	Patient Transition	0.7584	0.7584	0.7545	0.7545	0.7594	0.7594
0.97		0.9698	0.9698	0.9698	0.9698	0.9699	0.9699
0.66	Normal Transition	0.6300	0.6300	0.6562	0.6562	0.6867	0.6867
0.92		0.9173	0.9173	0.9217	0.9217	0.9289	0.9289

### Test on real data

To demonstrate the utility and further validate the performance of BIMMER, we applied BIMMER to a real dataset published in [4], which includes MBDCap-seq reads of whole genome methylation profiles from 10 normal and 75 breast cancer tissues from the 1000 methylome project (http://cbbiweb.uthscsa.edu/KMethylomes ). The raw reads (FASTQ) file of MBDCap-seq data was first aligned to UCSC hg18 genome by BWA aligner [17]. The aligned SAM file was then converted to BED format later for further analysis.

The initial model parameters of the EM algorithm are defined in Table 3. Table 4 shows the estimated parameter set of the second hidden layer. The weight  α was predicted to be 0.3519, which means the transition probability A**_n _**possesses about 35.2% of influence while the conditional probability $P\left(n_{t}|d_{t},c_{t}\right)$ has about a weight of 64.8% on the state of *n_t_*.

**Table 3:** Initial values and the prior probabilities of BIMMER.

$m_{n}$	1	0			$m_{c}$	1	0			dm	1	0			State	$m_{c}$	$m_{n}$	dm
1	0.9	0.1			1	0.9	0.1			**1**	0.9	0.1			**0**	0.1	0.1	0.9
0	0.1	0.9			0	0.1	0.9			**0**	0.1	0.9			**1**	0.9	0.9	0.1

**Table. 3-1**					**Table. 3-2**					**Table. 3-3**					**Table. 3-4**			

Table. 3-1 to Table. 3-3 are initial transition probabilities for $m_{n}$, $m_{c}$ and dm; Table. 3-4 enlists the initial probabilities of $m_{n}$, $m_{c}$ and dm.

**Table 4:** The estimated parameters of the second hidden layer

$\tau_{dm}$	$A_{dm}$		**Weight ** α
**0.99999**	0.9705	0.0295	0.3519
**0.00001**	0.2862	0.7138	

**Figure 4:**
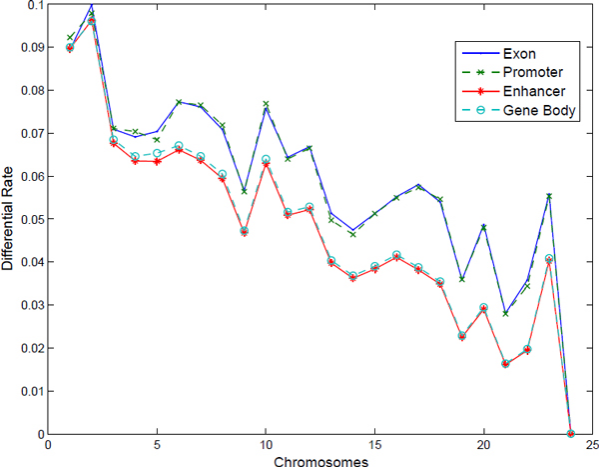
**Differential rate of 4 types of genomic regions in different chromosomes**.

Among the entire genome, about 8.83% of the bins were detected with differential methylation. Among these differential methylated bins, 95.6% of them are hypo-methylation (less degree of methylation in cancer), while only a minority of bins (4.4%) presented hyper-methylation (more degree of methylation in the cancer samples). Genome-wide differential rates on 4 regions (promoter region (±2kbp of transcription start position), enhancer region (100kbp after transcription end position), exons region and gene body) are plotted in Figure 4, where the detailed differential rates of the 4 regions in the 24 chromosomes are shown in Table 5. As expected, the promoter region and the exon possess higher differential methylation rate than the enhancer regions and gene body. Interestingly, chromosomes 1-2 have a significantly higher differential methylation rates in 4 genomic regions than those regions in other chromosomes.

**Table 5:** Differential rate of 4 regions on 24 chromosomes

Chromosome	Promoter	Exon	Enhancer	Gene Body
Chr1	0.005729	0.015478	2.331E-4	3.890E-4
Chr2	0.003440	0.009209	1.528E-4	2.357E-4
Chr3	0.001957	0.006006	9.138E-4	1.402E-4
Chr4	0.002017	0.005697	8.264E-5	1.336E-4
Chr5	0.001348	0.004764	9.434E-5	9.545E-5
Chr6	0.001857	0.005404	1.131E-4	1.300E-4
Chr7	0.002172	0.005126	1.022E-4	1.287E-4
Chr8	0.002223	0.004775	8.555E-5	1.182E-4
Chr9	0.001470	0.003957	6.925E-5	1.079E-4
Chr10	0.001583	0.004071	6.994E-5	1.044E-4
Chr11	0.001412	0.003576	6.113E-5	9.748E-5
Chr12	0.001212	0.003218	5.683E-5	8.236E-5
Chr13	0.001256	0.002956	5.161E-5	7.356E-5
Chr14	0.001070	0.002745	4.777E-5	6.970E-5
Chr15	0.001162	0.002940	4.717E-5	7.995E-5
Chr16	0.001172	0.002930	5.095E-5	8.412E-5
Chr17	0.001196	0.002969	5.242E-5	8.350E-5
Chr18	0.001090	0.002745	4.886E-5	7.272E-5
Chr19	8.860E-4	0.002388	4.385E-5	6.917E-5
Chr20	0.001014	0.002686	5.052E-5	7.976E-5
Chr21	9.763E-4	0.002416	4.783E-5	6.888E-5
Chr22	9.368E-4	0.002513	4.462E-5	7.624E-5
ChrX	6.024E-4	0.001642	2.793E-5	4.002E-5
ChrY	4.400E-4	0.001178	2.086E-5	2.979E-5
Total	0.001225	0.003201	5.644E-5	8.452E-5

**Figure 5:**
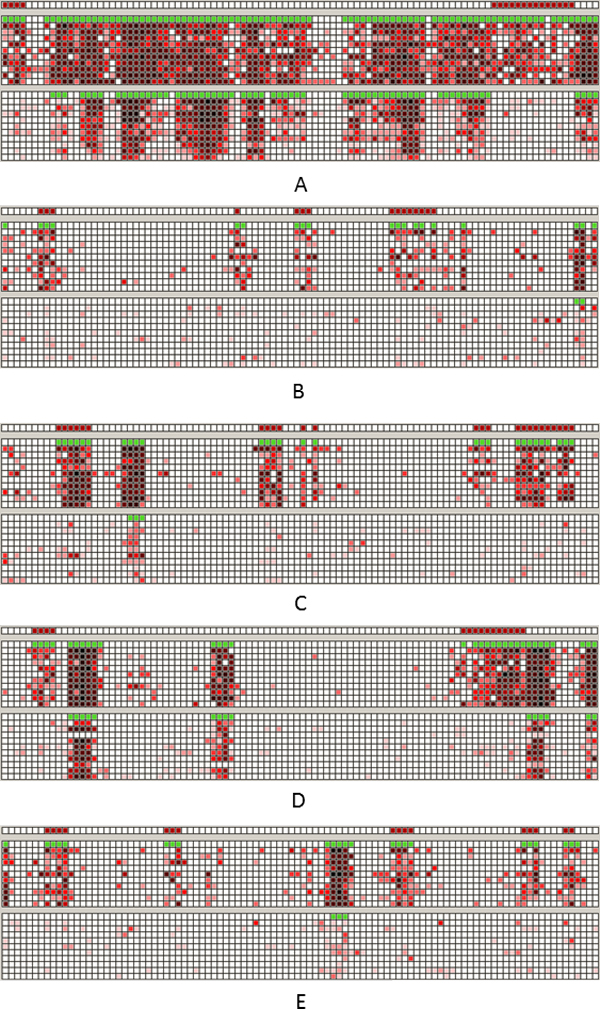
**Density Plots of Breast Cacner Related Differentially Methylated Genes A**. Density Plot for ACADL (Chr11:1,986,988-1,996,988). B. Density Plot for DAB2IP (Chr20:42,346,800-42,356,800). C. Density Plot for IL6 (Chr18:5,228,722-5,238,722). D. Density Plot for IMPACT (Chr9:2,150,455-2,160,455). E. Density Plots for RARA (Chr7:127,223,462-127,233,462). For each sub-figure, the plot includes 3 panels. The top panel shows a single line of squares, each representing a predicted differential methylation at a bin, where red square denotes differentially methylation. The second panel shows the reads density of 10 normal samples together with the predicted methylation status (the top indicator line). The reads density is in red color and color intensity is proportion to the read counts. The green square in the indicator line denotes that the bin is predicted to be methylated. The third panel shows the read density of 10 breast cancer patients and the corresponding predicted methylation status.

Next the genome-wide methylation information was mapped to individual genes to determine whether a gene is differential methylated in the cancer samples vs. the normal samples. For this mapping, 17814 gene symbols were selected (TCGA-BRCA entry). The location information of these gene symbols were downloaded and mapped to the bin location. To avoid possible false positive, a permutation test was conducted on the predicted methylation result to obtain the prediction p-value and a 0.05 significant level was applied. Among this 17814 genes, 293 genes (additional file 1) were detected with significant differential methylation. The methylation status was very similar to that of the genome-wide result, where among these 293, only 4 genes were hyper-methylated and the rest are all hypo-methylated. Table 6 listed the top 20 methylated genes according to their differential methylation rates. The first ranked gene is CDC5L, which encodes the cell division cycle 5-like protein. [18,19] Research showed that this gene is highly involved in the RNA-splicing and could be a target for cancers [20,21]. The next 16 genes that shared the same differential methylation rate include BCL3, which is highly involved in breast cancer metastasis and tumor progression [22-24], c6orf123 and c6orf124, two RNA genes which have been showed to be associated with ovarian cancer, CRYAB, a tumor suppressor gene [25], CRIP2, a gene that encodes the cysteine rich intestinal protein 2 and has been implicated to have effect on suppressing tumorigenesis [26], HSD17B1 gene that produces an enzyme that catalyzes the conversion of esrone to estradiol, and is hypothesized to influence endometrial and breast cancer risk[27,28], and PTPN12, which has been shown to be involved in the ovarian cancer and breast cancer and is also survival related [29-31]. Over all, a lot of top ranked differential methylated genes show associations with breast cancer or other cancers. In addition, the differential methylation status of three sets of breast cancer genes including six survival related genes, 8 tumor size related genes and eight ER+ related genes (Table 7) were examined. For the six survival related genes, 3 out of five were detected with significant differential methylation. In contrast, both tumor size related and ER+ gene sets have about 25% differential methylation rate. The differential methylation density maps of a subset of these genes were also shown in Figure 5. The density clearly confirms that BIMMER has correctly identified the differential methylation regions and the advantages of the HMM model for differential methylation analysis is clearly shown. When a bin having similar reads counts in cancer and normal sample sits in the middle of a stretch differential methylated region (Figure 5.A. second DMR region; Figure 5.C last DMR region), it will be predicted differential methylation by BIMMER because BIMMER considers correlation between adjacent bins. This gives BIMMER the ability to avoid possible false negative predictions.

**Table 6:** Top 20 differential methylated gene

GENE SYMBOL	DIFFMETHY RATE	METHYLATION STATUS
**CDC5L**	0.380952381	0
**BCL3**	0.333333333	0
**C6ORF123**	0.333333333	0
**C6ORF124**	0.333333333	0
**COX6B1**	0.333333333	0
**CRYAB**	0.333333333	0
**GRIP2**	0.333333333	0
**HSD17B1**	0.333333333	0
**NAGLU**	0.333333333	0
**OR5M11**	0.333333333	0
**PHTF2**	0.333333333	0
**PIH1D2**	0.333333333	0
**PTPN12**	0.333333333	0
**RSBN1L**	0.333333333	0
**SFTPD**	0.333333333	0
**TAAR6**	0.333333333	0
**TAAR8**	0.333333333	0
**C6ORF192**	0.317460317	1
**AKR1C4**	0.285714286	0
**APOC2**	0.285714286	0

**Table 7:** Differential rate of normal and patient samples for 22 breast cancer related genes

Gene Name	Relation with Breast Cancer	Differential Methylation Status	Differential Rate
**RECK**	Related to Survival	Yes	0.2195
**SFRP2**	Related to Survival	No	
**ITR**	Related to Survival	Not maped	
**UGT3A1**	Related to Survival	No	
**ACADL**	Related to Survival	Yes	0.3659
**UAP1L1**	Related to Survival	Yes	0.2195
**HSD17B12**	Related to Tumor Size	No	
**IMPACT**	Related to Tumor Size	Yes	0.2683
**IL6**	Related to Tumor Size	Yes	0.3171
**PLAT**	Related to Tumor Size	No	
**NCL**	Related to Tumor Size	No	
**FES**	Related to Tumor Size	No	
**PLAUR**	Related to Tumor Size	No	
**ALK**	Related to Tumor Size	No	
**IRF7**	Related to ER+	No	
**RARA**	Related to ER+	Yes	0.2195
**ACG2**	Related to ER+	No	
**AXL**	Related to ER+	No	
**ZNF264**	Related to ER+	No	
**DAB2IP**	Related to ER+	Yes	0.1951
**FZD9**	Related to ER+	No	
**SRC**	Related to ER+	No	

## Discussion and future work

In this work, BIMMER, an HMM based algorithm for DMRs detection for MBDCap-seq data is proposed. BIMMER models the methylation status and differential methylation status simultaneously, which does not suffer from the error propagation of existing two-step DMRs detection algorithms. In addition, BIMMER can handle replicate samples at the same time, producing more coherent detections. BIMMER relies on an EM algorithm to estimate the model parameters jointly. BIMMER was validated using simulated data and applied to real breast cancer datasets.

In the future work, four possible aspects could contribute to the performance improvement of BIMMER. First, adding more states of differential methylation into the HMM model and including hyper- and hypo- methylation type status will clearly provide better interpretation of the result. Second, more accurate models can be developed to model the differential methylation status and methylations in different phenotype of samples. Third, more accurate solution could be introduced to replace the weighted average approach. For example, product of experts (PoE) [32] has been shown to be a power tool in recent studies. Finally, more epigenetic information such as CpG island or histone modification can be included into BIMMER to produce biologically more relevant results.

## Competing interests

The authors declare that they have no competing interests.

## Authors' contributions

ZM and CM designed the method and drafted the manuscript. TH provided the data. YC helped with preprocessing the data. YC and YH supervised the work, made critical revisions of the paper and approved the submission of the manuscript.

## Supplementary Material

Additional File 1 **List of significantly differentially methylated genes reported by BIMMER**. Differential methylation rates and methylation status are also provided. Click here for file

## Declaration

Publication of this article has been funded by NSF Grant (CCF-1246073) to YH and a Qatar National Research Fund(09-897-3-235) to YH and YC to support this project. This work also has been supported by the help of UTSA Computational System Biology Core, funded by the National Institute on Minority Health and Health Disparities(G12MD007591) from the National Institutes of Health.

This article has been published as part of *BMC Bioinformatics *Volume 15 Supplement 12, 2014: Selected articles from the IEEE International Conference on Bioinformatics and Biomedicine (BIBM 2013): Bioinformatics. The full contents of the supplement are available online at http://www.biomedcentral.com/bmcbioinformatics/supplements/15/S12.
